# A Quick Guide for Building a Successful Bioinformatics Community

**DOI:** 10.1371/journal.pcbi.1003972

**Published:** 2015-02-05

**Authors:** Aidan Budd, Manuel Corpas, Michelle D. Brazas, Jonathan C. Fuller, Jeremy Goecks, Nicola J. Mulder, Magali Michaut, B. F. Francis Ouellette, Aleksandra Pawlik, Niklas Blomberg

**Affiliations:** 1 Structural and Computational Biology (SCB) Unit, European Molecular Biology Laboratory (EMBL), Heidelberg, Germany; 2 The Genome Analysis Centre (TGAC), Norwich Research Park, Norwich, United Kingdom; 3 Ontario Institute for Cancer Research, MaRS Centre, West Tower, Toronto, Ontario, Canada; 4 Heidelberg Institute for Theoretical Studies (HITS) gGmbH, Heidelberg, Germany; 5 The Computational Biology Institute, George Washington University, Innovation Hall, Virginia, United States of America; 6 Computational Biology Group, Institute of Infectious Disease and Molecular Medicine (IDM), University of Cape Town Faculty of Health Sciences, Cape Town, South Africa; 7 Computational Cancer Biology, Netherlands Cancer Institute, Amsterdam, the Netherlands; 8 Department of Cell and Systems Biology, University of Toronto, Toronto, Canada; 9 The Software Sustainability Institute, School of Computer Science, University of Manchester, Manchester, United Kingdom; 10 ELIXIR Hub, Wellcome Trust Genome Campus, Cambridge, United Kingdom; George Mason University, UNITED STATES

## Abstract

“Scientific community” refers to a group of people collaborating together on scientific-research-related activities who also share common goals, interests, and values. Such communities play a key role in many bioinformatics activities. Communities may be linked to a specific location or institute, or involve people working at many different institutions and locations. Education and training is typically an important component of these communities, providing a valuable context in which to develop skills and expertise, while also strengthening links and relationships within the community. Scientific communities facilitate: (i) the exchange and development of ideas and expertise; (ii) career development; (iii) coordinated funding activities; (iv) interactions and engagement with professionals from other fields; and (v) other activities beneficial to individual participants, communities, and the scientific field as a whole. It is thus beneficial at many different levels to understand the general features of successful, high-impact bioinformatics communities; how individual participants can contribute to the success of these communities; and the role of education and training within these communities. We present here a quick guide to building and maintaining a successful, high-impact bioinformatics community, along with an overview of the general benefits of participating in such communities. This article grew out of contributions made by organizers, presenters, panelists, and other participants of the ISMB/ECCB 2013 workshop “The ‘How To Guide’ for Establishing a Successful Bioinformatics Network” at the 21st Annual International Conference on Intelligent Systems for Molecular Biology (ISMB) and the 12th European Conference on Computational Biology (ECCB).

## Introduction

In many cases, bioinformatics communities play a central role in the success of highly complex scientific projects and consortia. This guide was inspired by a workshop, “The ‘How To Guide’ for Establishing a Successful Bioinformatics Network,” that took place as part of the ISMB/ECCB 2013 conference. During the workshop, organizers, presenters, and panelists shared insights gained from participating in a range of successful bioinformatics communities with an audience of more than 100 additional participants. One session of the workshop was an interactive, small-group discussion in which all participants described their opinions on the benefits and disadvantages of contributing to bioinformatics communities. The opinions and ideas presented in this guide are a synthesis of the experiences and opinions contributed by all participants of the workshop.

## Communities, Networks, and Organizations

While the terms “communities,” “networks,” and “organizations” are sometimes used interchangeably, in other contexts they refer to distinct kinds of social structures. This guide concerns, in part, interactions between these different structures. Thus, to avoid confusion, we begin by defining our use of these terms in this article.

Borrowing from anthropology and learning science, as well as management and organizational behaviour studies, we define “network” as a set of “relationships, personal interactions, and connections” [1] between a group of people and “community” as a group of people possessing a shared identity around a topic or set of challenges of joint interest, linked to a collective intention of working together to build knowledge and solutions around this topic or challenges (see also *The Art of Community* by Jono Bacon [2]). We also define “organization” as an organized or cohesive group of people working together to achieve commonly agreed goals and objectives, in which specific roles and responsibilities are openly acknowledged for some or all members of the group. This explicit acknowledgment of specific roles for members of the group is often linked to the establishment of the group as a legal entity [3].

A successful community or organization is one that effectively and efficiently achieves its goals; such success is easier to assess in the context of explicitly stated goals. Working with the definitions above, explicit goals and missions are typically associated with organizations rather than with communities. Thus, it is typically organizations associated with communities, rather than communities themselves, that are identified as successful.

## Benefits of Participating in Bioinformatics Communities: Opportunities for Collaborative Research, Professional Development, Education, and Training

You are almost certainly a member of one or more professional networks or organizations, for example, professional societies such as the International Society for Computational Biology (ISCB) [4], scientific consortia such as the Encyclopedia of DNA Elements (ENCODE) [5], social media networks such as LinkedIn or ResearchGate, or the groups and institutions associated with your workplace (the group, the institute, the department you work in). By actively engaging and collaborating with others in these networks and organizations, on topics of mutual interest, you are also a member of one or more professional communities.

Scientists participating in research-driven communities have delivered a wide range of high-impact publications and life science data sets. These have typically been associated with projects (time-boxed, funded activities with agreed outcomes) such as ENCODE [5], the Human Microbiome Project [6], Galaxy [7,8], Bioconductor [9], BioJS [10], and the Virtual Liver Network [11,12].

Recent analyses highlight the increase in the proportion of science carried out in such communities of collaboration, and also the increase in the size of these collaborations, particularly when considering international collaborations [13–16]. This increase in the importance of collaborative science has even spawned its own field of social science research, the Science of Team Science (SciTS) [17–21].

Beyond these research-project-focused communities, other common bioinformatics-related activities are carried out in communities including professional/career development (especially training and education) or tool and resource development. Examples of such development-focused communities include interactions between groups of fellows and students (e.g., ISCB Student Council and Regional Student Groups [RSGs] [22] or the Software Sustainability Institute's [SSI] [23] Fellowship Program http://www.software.ac.uk/fellowship-programme), local geographically-focused groups of bioinformaticians (e.g., TorBUG http://www.torbug.org or Heidelberg Unseminars in Bioinformatics [HUB] [24] http://www.hub-hub.de), bioinformatics trainers and educators (e.g., GOBLET [25]), heads of nodes and technical coordinators involved in bioinformatics infrastructure development and capacity building (e.g., ELIXIR http://www.elixir-europe.org or H3ABioNet http://www.h3abionet.org), and cooperations between software/tool developers and their larger user communities (e.g., Bioconductor [9] or Galaxy [7,8,26]).

Communities focused around a common set of knowledge, skills, and tasks (“communities of practice” [27–29]), such as the bioinformatics communities discussed in this guide, have been recognized as crucial for training, development, and education. Bioinformatics communities provide a forum for participants to share information and experiences with each other, to learn from each other, and to develop themselves professionally. More formally, training events and activities delivered by bioinformatics communities are opportunities both to share expertise and knowledge within the community and to promote and strengthen the interactions (between and within trainers and trainees) that are the basis of successful communities.

## Different Kinds of Communities

Communities can be classified as “bottom-up” (or “grassroots”) or “top-down.” A “bottom-up” community is established by a group of people who find each other through their desire to collaborate around a topic of shared interest, typically with little or no funding or support from larger communities or organizations and little or no formal structure (although, as described by Jo Freeman in her essay on the inherent structures of groups that claim to be “unstructured,” there will inevitably be some form of structure, with an associated hierarchy, within these organizations [30]). An example of such a community, Heidelberg Unseminars in Bioinformatics (HUB) [24], was conceived through informal discussions between people from several Heidelberg organizations who were interested in bioinformatics and exploring alternative meeting formats. HUB participants collaborate together as volunteers, in a relatively informal fashion, via regular local meetings on these topics (unconferences or unseminars [31]). HUB recently established an organization linked to HUB and is currently in the process of establishing itself as a legal entity. This move towards formalizing the community into an organization was motivated by issues of liability limitation, the benefits of establishing clarity of goals and structure described elsewhere in this guide, and the opportunities it provides for setting up an organizational bank account and being eligible to apply for funding. Other examples of such “bottom-up” organizations described in this guide include the pan-Canadian Bioinformatics User Groups (BUG) of VanBUG, TorBUG, and MonBUG organizations and the ISCB Student Council and Regional Student Groups [22]. Guidelines for starting new communities of this kind have been described based on the experience of establishing more than 20 different RSGs [32].

"Top-down" communities linked to organizations are typically created as a result of a strategic decision. The goals of such communities are typically goals shared by the founding stakeholders. A major challenge faced in establishing such organizations is to reach agreement between large numbers of stakeholders on the common goals, costs, and expected return on the investments. A further challenge is to find ways to effectively foster collaborations across geographical and organizational boundaries. ELIXIR (www.elixir-europe.org), the European infrastructure for biological information, is an example of such a “top-down” organization, growing out of a community effort. In 2007, a consortium of bioinformaticians across Europe were awarded European Union (EU) funding to plan and prepare the building of a trans-European infrastructure to support the transfer, storage, and analysis of biological information. After more than six years of intense planning and high-level political discussions with science funders and potential member states, ELIXIR was established through an agreement between its member states in December 2013. Thus, ELIXIR represents the evolution from: a shared idea of a group of researchers to a large, funded, project to a transnational organization with a set of clearly defined goals, organizational governance structures, legal framework, and funding processes. Members of the community linked to ELIXIR are bioinformaticians involved in service and infrastructure delivery across Europe, collaborating together to “orchestrate the collection, quality control and archiving of large amounts of biological data produced by life science experiment.”

Communities can also be classified according to their progression through a “community lifecycle” [33]. Millington [34] provides specific advice on building a community during different phases of the lifecycle. In particular, he focuses on a shift from an initial phase where most activity, and new members, comes from the activity of the founders of the community and/or a community manager to a more mature phase where community growth and activity is driven by the activities of others. This change is accompanied by a shift in the importance for founders and community managers from more tactical, hands-on, one-to-one activities within the community to more macro-level, strategic activities. This often necessitates a formalization of the community objectives and processes, e.g., through the introduction of formal code reviews and release processes or introduction of subgroups focusing on specific tasks or deliverables.

## A Quick Guide to Building a Successful Bioinformatics Community

Analyses of successful, high-impact collaborations, organizations, networks, and communities have identified several common features of such groups including effective interactions, communication, and leadership; a clear, shared vision of the aims of the group; and a passionate commitment by participants to the goals of the group [2,34–38]. To gain further insight into features of highly successful bioinformatics communities, we hosted a workshop on bioinformatics communities at the ISMB/ECCB 2013 meeting in Berlin. Based on the discussions and presentations featured in the workshop, we provide a “Quick Guide” of practical actions you can take to build and develop successful bioinformatics communities. Where relevant, we describe examples provided by the participants of the workshop to reinforce these points.

As described above, there is considerable diversity in bioinformatics communities, for example, in terms of size, funding, shared interests and goals, influence, organizational structures, and maturity. However, despite this diversity, the points included in this guide are, we believe, relevant and beneficial to the success of any such community.

### 1. Ensure membership in a community brings obvious benefits to its members

People choose to be part of a community because they perceive that they will benefit from this participation. This could range from simply enjoying the company of collaborators within the community to providing opportunities to participate in projects with a direct benefit to professional development (e.g., contributing to high-impact publications). Making these benefits clear motivates newcomers to join the community and existing members to continue (and perhaps increase) their levels of participation in the community. An example of this can be seen in the activities of the ISCB Student Council and Regional Student Groups [22]. They strongly emphasize (in their communications with members and the wider public) that participating in the activities of the organization brings clear benefits in terms of opportunities for training, gaining experience of leadership and organizing events, and building a stronger professional network. The clarity of these benefits to existing members of these organizations is described as essential for their success by creating a core group of highly motivated, competent members who work hard to achieve the mission of the organization.

### 2. Provide a description of the goals, vision, and mission of the community that are accessible, clear, and concise

A clear, concise set of goals describing the aims of a community is invaluable for communicating the focus and purpose of the mission to members of the community, and to other stakeholders. This can, for example, be very useful for helping potential members decide whether or not their interests align with those of the community. By providing a context to assess the utility and appropriateness of community activities, a description of the goals, vision, and mission of the community can also be a useful aid for setting priorities and making decisions within the community, i.e., when trying to choose between several alternative courses of action, the best decision would be the one that helps the community best achieve its goals.

Note that, as mentioned in the introduction, explicit goals and missions are typically associated with a specific organization, rather than the community linked to the organization. Writing and agreeing on these can be a major challenge, particularly for “top-down” organizations, as this process will involve prioritization and trade-off between funders and other stakeholders.

The mission of the ISCB Student Council [22], for example, is to “promote the development of the next generation of computational biologists.” It is a clear mission, attainable and of great perceived value to its members. A key factor for the success of the Bioconductor project [9] was described as being the clear, shared vision for the direction and methods of software development, including emphasis on high-powered statistics and commitment to common interfaces and containers. The importance of a shared, clearly described mission was also described as important for the success of ELIXIR, whose mission is “to build a sustainable European infrastructure for biological information, supporting life science research and its translation to medicine and the environment, the bio-industries and society.”

### 3. Facilitate communication between members of the group

Communication is an essential component of any collaboration. One cannot collaborate without communication. Therefore, a successful community must be built around effective communication.

Different means of communication are better suited to different purposes. For example, announcing upcoming events to a large, geographically distributed community is well suited to electronic communication and social media such as email, Twitter, LinkedIn, wikis, and other online resources. With minimal effort, these means of communication can easily deliver information to many different people. In contrast, discussions of complex, urgent issues involving input from many participants are typically best carried out in face-to-face meetings. Thus, communication within a community is typically a mixture of face-to-face meetings and distant/remote/electronic communication.

It is important to be aware that people have different preferences for communication. Some people are email enthusiasts, but refuse to use social media tools such as LinkedIn, Twitter, or Facebook, while others may find it intimidating to edit a wiki. Thus, it may be useful to communicate using a range of different media and challenges. At the same time, the more modes of communication you use, the more resources are required. If communication appears to be effective within your group, it may be best to avoid introducing additional communication channels that take more resources to support and use than they bring benefit to the group.

All participants in the workshop emphasized the importance of effective, regular communication for the success of a community. The Galaxy project [7,8,26] provides an excellent example of this, using a range of different ways in which community members can communicate with each other, including a substantial wiki, Twitter, mailing lists, a custom Biostar [39] forum (https://biostar.usegalaxy.org), regular face-to-face meetings, and conference calls. Open, wide-reaching communication is also important in the project for acknowledging and publicizing contributions to the project, in particular using social media. The importance of community building and outreach for Galaxy is reflected in the allocation of approximately half of the project’s budget to these activities. In a similar way, the success of the Bioconductor project [9] is also linked to its lively mailing list, many face-to-face developer meetings, and regular interactions between users and developers at training courses.

### 4. Establish and communicate a clear, transparent organizational structure

Even groups that claim to be “unstructured” have a structure. This structure might be an equal distribution of power between all members or an unequal distribution of power between different members together with the existence of (unacknowledged) subgroups (cliques) of stronger relationships within the group [30]. Such unacknowledged power structures are non-transparent, difficult to identify and understand, and tend to promote distrust within the group. Thus, it is strongly recommended that an organization linked to, or representing, a community, has a clearly defined, transparent, leadership structure that is communicated to all members of the community. This helps everyone to understand which responsibilities and decision-making powers are held by which members of the organization. A clear decision-making structure can also help reduce the time needed to make important decisions.

By considering the role and presence of structure in communities, we highlight a key difference between a community and an organization linked to that community. Explicit structures of this kind are typically associated with organizations rather than the communities associated with these organizations (as the word suggests, organizations are more “organized” than communities). Thus, in this section of the article, we focus on the structure of organizations strongly linked to specific bioinformatics communities.

Different organizations have been successful with different kinds of organizational structures [40,41]. For relatively small, low-resource organizations, such as HUB, a very simple structure has been successful, with a board consisting of three members, all other members having the same organizational status. At the other end of the spectrum, ELIXIR, where national states rather than individuals or organizations are the formal members, has a structure with a clear division of tasks, assigned accountabilities, and the level of governance and oversight necessary for transnational collaboration. The ELIXIR operational structure consists of a central, jointly funded Hub linked to nodes in the member states. The governing structure is made up of the ELIXIR Board (with representation from national science funders or ministries) with operational and scientific responsibility provided by the ELIXIR director together with national node directors through the “Heads-of-Nodes” committee.

Several speakers in the workshop that inspired this article highlighted the importance of a clear management structure for the success of communities, including the H3ABioNet project and the ISCB Student Council and Regional Student Groups [22]. A clear structure, and, importantly, assigned roles and responsibilities, provides a framework for collaboration. Forming subgroups with clearly assigned tasks and responsibilities allows the individual contributors to focus on their activities without feeling pressured to consult widely before implementation.

At a more operational level, clear rules for submitting code to Bioconductor [9] are described as important for the project’s success [42]. These rules, written by core developers of the project, facilitate the integration of contributions from diverse developers into the Bioconductor code base, while clear communication of the guidelines to potential code contributors helps manage expectations and reduce the wasted efforts by contributors. This is also an example of the use of authority, in this case of the leadership of the Bioconductor project, to facilitate and coordinate the activities of the community; if the leadership were not prepared to insist on this set of rules, the rate of growth, and utility of the project, would be expected to suffer.

### 5. Whenever possible, make your communications open and transparent

Open communication is important for building trust within the group. Giving everyone access to the information on which decisions have been made, and for what reasons, goes a long way to avoiding misunderstandings and distrust amongst community members.

The Bioconductor project [9] is strongly focused on a shared vision of open source and open development, both of which make a major contribution to the transparency and openness of interactions within the project. Openness and transparency in reporting structure was also emphasized as important for the ISCB Student Council and RSGs [22]. An example of the policy and utility of such openness is HUB [24], where the minutes and agendas of all planning and other meetings associated with the project are posted for all to read on the HUB wiki.

### 6. Make it easy and enjoyable to participate in the activities of the community

Participation increases the amount of activity of the community, and at the same time fosters a sense of ownership that is a key motivator for further participation. An essential component of the success of the Bioconductor project [9] is that everyone is welcome and encouraged to participate in the project by contributing to discussions on mailing lists, code, code documentation, and joining courses and conferences linked to the project. The H3ABioNet project also makes a concerted effort to welcome and make it easy for a wide range of different people to contribute and remain active within the project, thus harnessing the good will and expertise available to the project and giving participants a feeling of ownership of and belonging to the network. The Galaxy project [7,8,26] also focuses considerable efforts on enabling and empowering the community to contribute to code, documentation, and discussions associated with the project. To achieve this, Galaxy and its web resources are designed to promote and facilitate sharing analyses, data, tools, and curation.

### 7. Acknowledge and highlight contributions to the community

If participants see that their work within a community is acknowledged and visible to all members of the community, and perhaps also to people outside the community, this can be a key motivator to further engage with the community. Openly acknowledging all contributions to Bioconductor [9] and Galaxy [7,8,26] code and resources was described as important for the success of the projects. Within the Galaxy project, a conscious effort is made to publicize and praise contributions, in particular using Bitbucket pull requests to integrate attributed software enhancements to the project’s codebase and a special “contributors” section in software release briefs. Small-group discussions during the workshop with all participants (not just organizers, speakers, and panelists) also described the provision of attribution/reward for contributions, particularly for junior researchers, as an important feature of successful communities.

### 8. Be aware that resources are essential to achieve the goals of a community

All activity expends resources; community activities are no exception. For example, organizing a TorBUG event requires many person-hours for planning the speaker and trainee sessions within an event, access to a web server to host the TorBUG website used for communicating with the community about the event, printed posters to advertise the event, a physical space to hold the meeting, refreshments and other consumables for use during the meeting, and laptops and a projector/beamer for presentations during the meeting. Of all of these, person-hours are of particular importance and value to the success of an event and thus the TorBUG community; without them, there would be no community.

Expending resources, particularly on facilitating and promoting communication, is therefore essential for achieving the goals of a community. Organizations seeking to promote community activity are thus strongly recommended to invest in establishing resources for the community. Part of this involves infrastructure and consumables (audiovisual [AV] equipment, computers, servers, stationary, web platforms), but, perhaps more importantly, it also involves providing funding for salaries for people committed to facilitating communications and other aspects of community growth and activity, i.e., funding a role of “community manager.” The Software Sustainability Institute [23], for example, uses significant resources to provide salary and other funding for a community leader position.

## Conclusion

We have established some general guidelines for building successful bioinformatics communities. Ever-improving access to higher-speed Internet, huge data production, and open source projects empower distributed international projects, which, in turn, feed into the development of new communities. In this article we describe the importance of openness and communication for the establishment and effective functioning of bioinformatics collaborations and communities. Perhaps our most important conclusion is that scientific communities are at the heart of many fulfilling bioinformatics-related careers. This highlights the importance for scientists of finding and participating in communities that align with their interests, goals, and values.

## Organizers, Presenters, and Panelists of the ISMB/ECCB 2013 Workshop “The ‘How To Guide’ for Establishing a Successful Bioinformatics Network”

The ideas, comments, and advice presented in this article are based on presentations and discussions from the ISMB/ECCB 2013 workshop “The ‘How To Guide’ for Establishing a Successful Bioinformatics Network.” These were carried out by people with experience in establishing, building, and maintaining a range of different bioinformatics communities and networks. To give context and provenance to these ideas, comments, and advice, we provide below the complete list of all organizers, presenters, and panelists from the workshop, with brief summaries of some of their activities in such networks and communities.

*Niklas Blomberg*: Director of ELIXIR. Leads ELIXIR, the European infrastructure for biological information. Has been an industry advisor in national eScience initiatives and an active participant in cross-industry research programmes.

*Aidan Budd*: Senior Project Manager for Bioinformatics at the European Molecular Biology Laboratory (EMBL) in Heidelberg, Germany. Manages an internal network for bioinformatics at EMBL in Heidelberg. Co-founder of the Heidelberg Unseminars in Bioinformatics series of events [24].

*Michelle Brazas*: Coordinator of the Canadian Bioinformatics Workshops (bioinformatics.ca) [43] and Manager of Bioinformatics Education at the Ontario Institute for Cancer Research. Involved in organizing the Toronto Bioinformatics User Group (TorBUG) networking and community-building events.

*Manuel Corpas*: Project Leader at The Genome Analysis Centre, UK. Technical coordinator of the ELIXIR UK node. Coordinator of the BioJS project [10]. Inaugural chair of ISCB’s Student Council [22].

*Jonathan Fuller*: postdoctoral fellow with Professor Rebecca Wade at the Heidelberg Institute for Theoretical Studies, in Germany. Member of the Virtual Liver Network [11,12]. Co-founding member of Heidelberg Unseminars in Bioinformatics (HUB) [24].

*Jeremy Goecks*: Assistant Professor of Computational Biology at George Washington University. A lead investigator for the Galaxy project [7,8,26], a Web-based platform for doing accessible, reproducible, and collaborative computational biomedical research.

*Wolfgang Huber*: group leader and senior scientist at the EMBL in Heidelberg, Germany. Long-term participant in the Bioconductor project [9]. Writer and maintainer of several software packages. Current member of the Bioconductor Advisory Board.

*Magali Michaut*: computational biologist in the Computational Cancer Biology (CCB) group of the Netherlands Cancer Institute. Involved in the ISCB Student Council since 2007 [22], served as elected secretary in 2009. Founded ISCB SC Regional Student Groups (RSGs [32]) in France and Europe.

*Nicola Mulder*: Associate Professor at the University of Cape Town heading the Computational Biology Group. Coordinator of H3ABioNet, a Pan African bioinformatics network for H3Africa [44]. President of the African Society for Bioinformatics and Computational Biology [45].

*Francis Ouellette*: Senior Scientist, Associate Director of the Informatics and Biocomputing platform at the Ontario Institute for Cancer Research (OICR); Associate Professor in the department of Cell and Systems Biology at the University of Toronto. Scientific coordinator and instructor with the Canadian Bioinformatics Workshop series (CBW—bioinformatics.ca) [43]. Involved in establishing Bioinformatics User Groups in Vancouver and Toronto (VanBUG.org and TorBUG.org). Advisor to SGD, Galaxy, GenomeSpace, HMP, H3ABionet, and Genome Canada.

*Aleksandra Pawlik*: Training Leader at the Software Sustainability Institute [23] supporting development of research software communities in various disciplines. Co-manages the Institute's Fellows' network.

*Reinhard Schneider*: Head of Bioinformatics Core Facility at the Luxembourg Centre for Systems Biology. Treasurer of the ISCB[4]. Actively involved in various international networks and advisory boards.
